# CircRNA_101505 sensitizes hepatocellular carcinoma cells to cisplatin by sponging miR-103 and promotes oxidored-nitro domain-containing protein 1 expression

**DOI:** 10.1038/s41420-019-0202-6

**Published:** 2019-07-29

**Authors:** Yanwei Luo, Yunfeng Fu, Rong Huang, Meng Gao, Fengxia Liu, Rong Gui, Xinmin Nie

**Affiliations:** Department of Blood Transfusion, the Third Xiangya Hospital of Central South University, Tongzipo Road 138, 410013 Changsha, China

**Keywords:** Cancer therapy, Tumour-suppressor proteins

## Abstract

Hepatocellular carcinoma (HCC) is one of the most common malignant tumors and a leading cause of cancer-related deaths worldwide. Emerging studies have shown that circular RNAs (circRNAs) are differentially expressed in HCC and play an important role in HCC pathogenesis and metastasis. However, the mechanism of circRNA in the chemoresistance of HCC remains unclear. In this study, we aimed to investigate the role of circRNA in cisplatin resistance of HCC. We identified a novel circRNA circRNA_101505 that was decreased in cisplatin-resistant HCC tissues and cell lines, and associated with a poor survival outcome. Gain-of-function investigations showed that overexpression of circRNA_101505 suppressed cancer cell growth in vivo and in vitro, and enhanced cisplatin toxicity in HCC cells. Mechanistic studies found that circRNA_101505 could sensitize HCC cells to cisplatin by sponging miR-103, and thereby promoting oxidored-nitro domain-containing protein 1 (NOR1) expression. In conclusion, the significant inhibitory effects indicate circRNA_101505 to be a potential therapeutic target for HCC treatment. Our findings provide significant evidence to further elucidate the therapeutic use of circRNA in HCC.

## Introduction

Hepatocellular carcinoma (HCC) is one of the most common malignant tumors and a leading cause of cancer-related deaths worldwide^1^. Owing to the high frequency of tumor metastasis, recurrence, and drug resistance, the survival rate of patients with HCC is low. Additionally, the effective therapies for advanced HCC patients are limited^2^. Thus, identifying novel targets for HCC therapy is urgently required. Emerging studies have found that circular RNAs (circRNAs) are differentially expressed in HCC and play an important role in HCC pathogenesis and metastasis^3^.

CircRNAs are a tissue-specific class of noncoding RNAs molecules. They are characterized by a covalently closed continuous loop without poly(A) tail^4^. Recently, thousands of endogenous circRNAs have been discovered. CircRNAs mediate gene expression by sponging microRNAs or interacting with other molecules and then inhibit their function^5^. For example, circRNA, circ-ZNF652 promotes migratory and invasive capabilities of HCC cells by sponging miR-203 and miR-502-5p and enhancing epithelial-mesenchymal transition (EMT)^6^. CircRNA circSETD3 was significantly downregulated in HCC tissues and cell lines and associated with unfavorable prognosis of HCC patients^7^. Matboli et al showed that the combination of hsa_circ_00156, hsa_circ _00224 and hsa_circ _00520 acted as biomarkers with higher sensitivities and specificities than alpha-fetoprotein in HCC^8^. Multidrug resistance occurs frequently in HCC during the long-term chemotherapy, leading to cancer relapse. An increasing number of studies have highlighted the key roles of ncRNAs, including miRNAs, long ncRNAs (lncRNAs) in chemoresistance of HCC^9^. However, the mechanism of circRNA in chemoresistance of HCC remains unclear.

In this study, we aimed to investigate the role of circRNA in cisplatin resistance of HCC. We identified a novel circRNA circRNA_101505, that was decreased in cisplatin-resistant HCC tissues and cell lines. Gain-of-function investigations showed that circRNA_101505 overexpression suppressed cancer cell growth in vivo and in vitro. Subsequent studies displayed that circRNA_101505 could sensitize HCC cells to cisplatin by targeting the miR-103/oxidored-nitro domain-containing protein 1 (NOR1) signaling axis.

## Results

### Downregulation of CircRNA_101505 is associated with cisplatin resistance in HCC

**Fig. 1:**
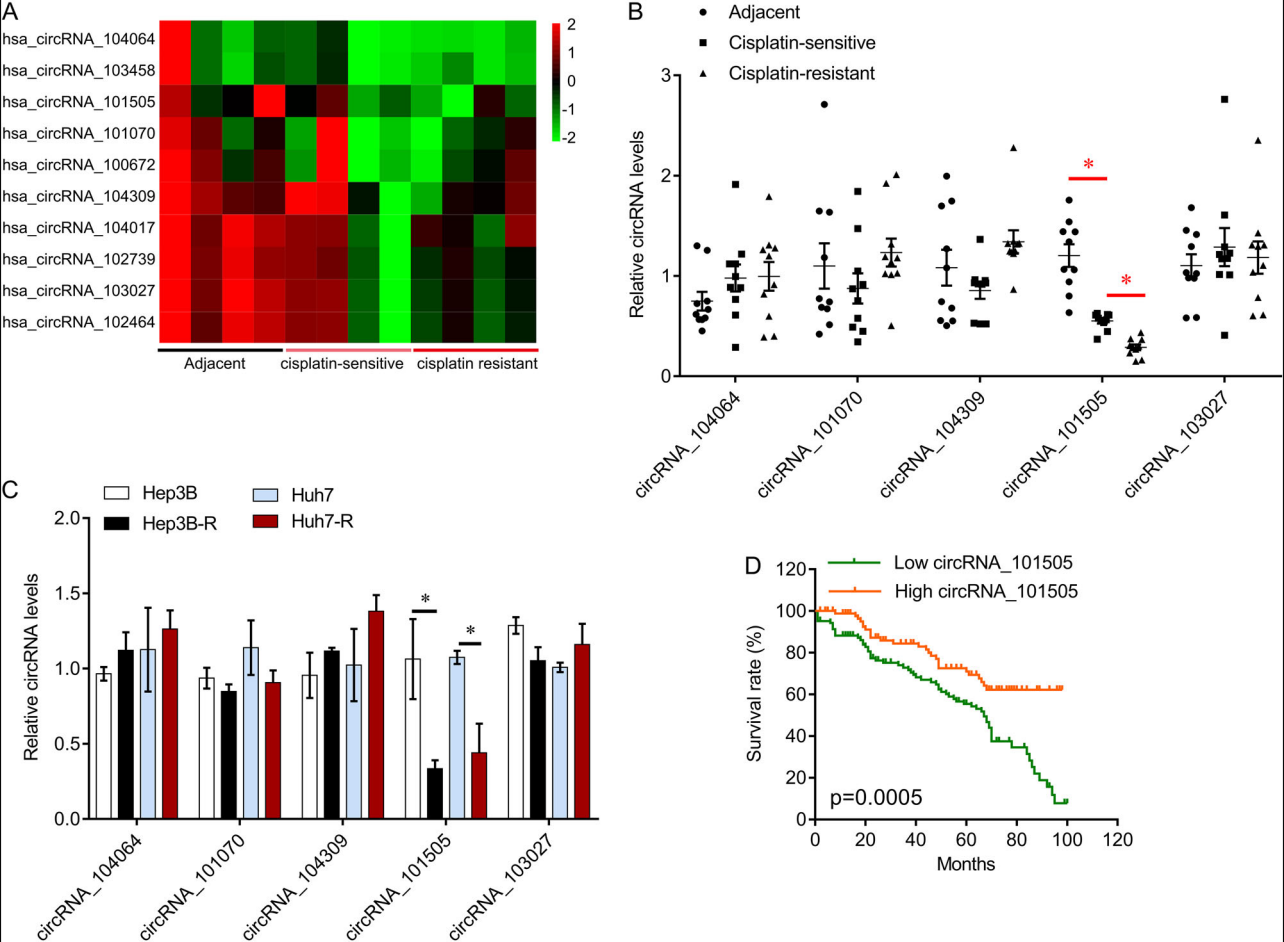
circRNAs expression in HCC. **a** The heatmap of deferentially expressed circRNAs in HCC. **b** QPCR was performed to confirm the expression of circRNAs in cisplatin-resistant and cisplatin-sensitive HCC tissues. **c** QPCR was performed to confirm the expression of circRNAs in cisplatin-resistant and cisplatin-sensitive HCC cell lines. **d** Kaplan–Meier curves of overall survival in HCC patients with low or high circRNA_101505 expression. *p < 0.05

To investigate the role of circRNAs in cisplatin-resistant HCC, we performed circRNAs array to identify the differentially expressed circRNAs. Several circRNAs were differentially expressed in cisplatin-resistant and -sensitive HCC (Fig. 1a). qPCR results further confirmed that circRNA_101505 was significantly decreased in HCC tissues compared with adjacent tissues, and its expression was lower in cisplatin-resistant HCC tissues than in the cisplatin-sensitive tissues (Fig. 1b). We also found that circRNA_101505 was significantly decreased in cisplatin-resistant HCC cell lines (Hep3B-R and Huh7-R) compared with the parental HCC cell lines (Fig. 1c). In addition, further survival analyses revealed that the HCC patients with low circRNA_101505 level had shorter overall survival than the patients had high circRNA_101505 level (*p* = 0.0005, Fig. 1d). These results suggest that circRNA_101505 is closely associated with cisplatin resistance in HCC.

### Overexpression of circRNA_101505 sensitizes HCC cells to cisplatin

**Fig. 2:**
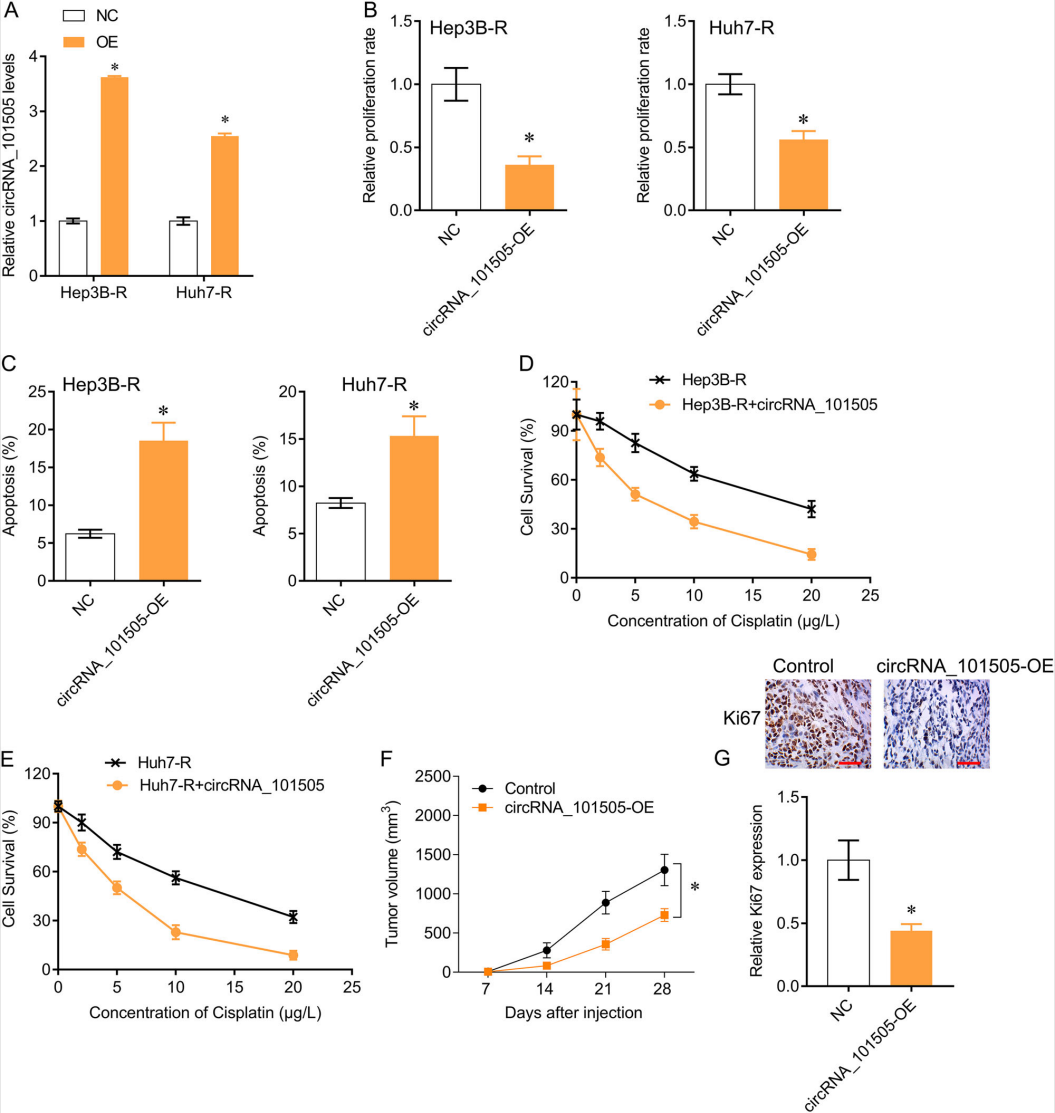
CircRNA_101505 sensitizes HCC cells to cisplatin. **a** QPCR for circRNA_101505 in Hep3B-R and Huh7-R cells after circRNA_101505 transfection. **b** Cell proliferation analysis showed that overexpressing circRNA_101505 inhibited proliferation of Hep3B-R and Huh7-R cells. **c** Flow cytometry showed that overexpressing circRNA_101505 induced apoptosis in Hep3B-R and Huh7-R cells. **d**, **e** Overexpressing circRNA_101505 sensitizes Hep3B-R and Huh7-R cells to cisplatin. (**f**) Hep3B-R cells that transfected with circRNA_101505 were injected into nude mice. The tumor volumes were measured every week. **g** Immunohistochemistry analysis for Ki67 in xenografted tumor tissues. Scale bar, 100 μm. *p < 0.05 vs control

To investigate the role of circRNA_101505 in cisplatin resistance in HCC, we overexpressed circRNA_101505 in Hep3B-R and Huh7-R cells (Fig. 2a). We found that overexpression of circRNA_101505 significantly inhibited cells proliferation (Fig. 2b) and induced apoptosis (Fig. 2c) in Hep3B-R and Huh7-R cells compared with negative control. In addition, circRNA_101505 upregulation sensitizes Hep3B-R and Huh7-R cells to cisplatin (Fig. 2d, e). Furthermore, the tumor suppressive effects of circRNA_101505 upregulation were also confirmed in vivo. Our results showed that the tumor volumes in nude mice injected with Hep3B-R cells expressing circRNA_101505 were smaller than in the control nude mice (Fig. 2f), and the proliferative marker ki67 expression was decreased in tumor tissues that expressing circRNA_101505 compared with control (Fig. 2g).

### CircRNA_101505 sponges miR-103 in HCC

**Fig. 3:**
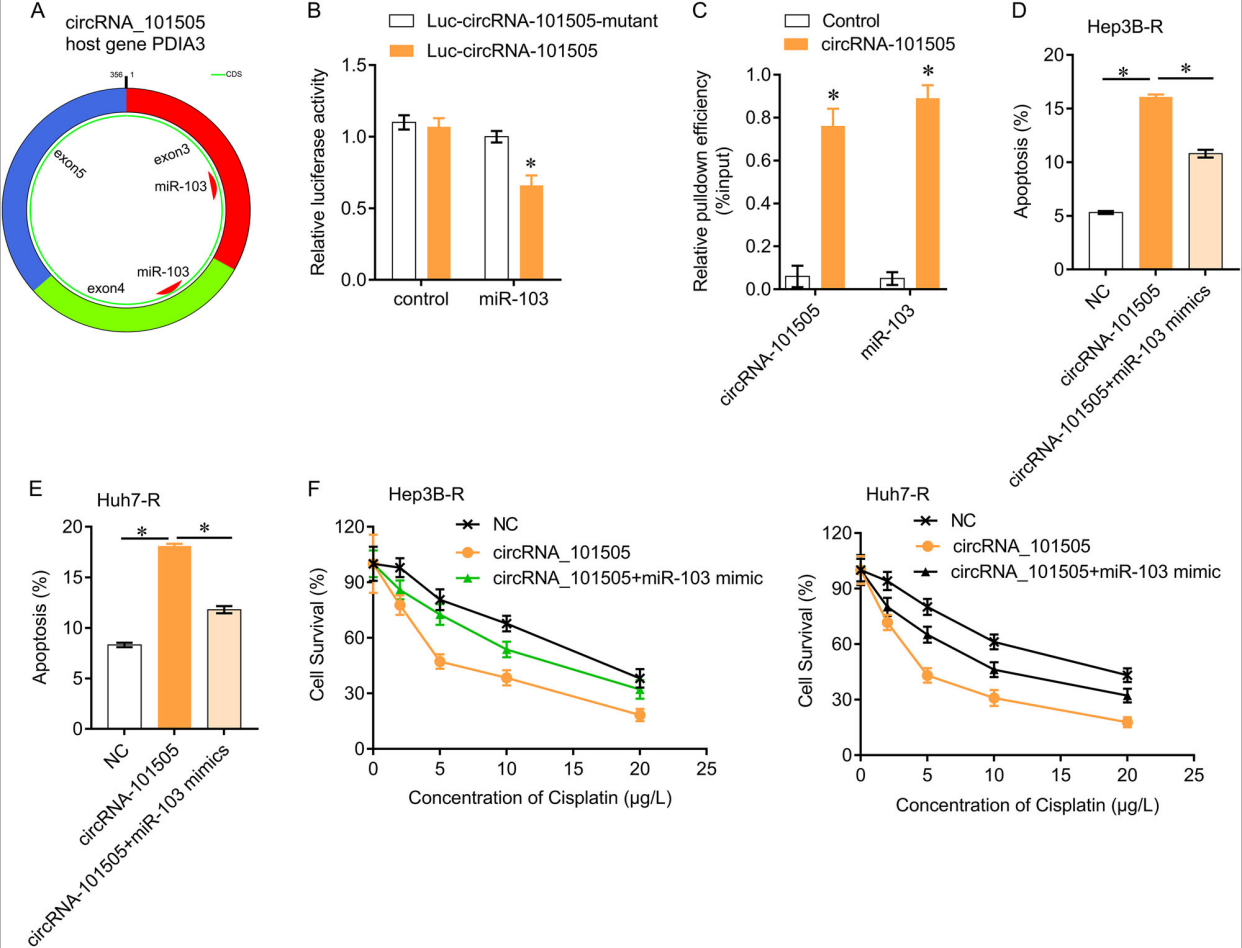
CircRNA_101505 serves as a sponge for miR-103 in HCC cells. **a** The predictive binding sites of miR-103 on circRNA_101505. **b** Hep3B-R cells were co-transfected Luc- circRNA_101505 or Luc- circRNA_101505 mutant with miR-103 mimics. Luciferase reporter gene assay was performed to measure luciferase activity. **c** RNA pull-down assay for the amount of circRNA_101505 and miR-103 in Hep3B-R cells. **d**, **e** circRNA_101505-induced apoptosis was reduced by miR-103 mimics in Hep3B-R and Huh7-R cells. (**f**) miR-103 mimics reversed circRNA_101505-enhance cisplatin toxicity in Hep3B-R and Huh7-R cells. *p < 0.05

We tested whether circRNA_101505 binds to miRNAs in HCC cells. The bioinformatics tool predicted two binding sites on circRNA_101505 for miR-103 (Fig. 3a). Further luciferase assay confirmed that circRNA_101505 targeted to miR-103 (Fig. 3b), suggesting that circRNA_101505 may function as a sponge to miR-103. In addition, RIP assay revealed that miR-103 directly interacted with circRNA_101505 (Fig. 3c). To investigate the downstream mechanism by which circRNA_101505 exerted its functions in HCC cells, the mimic of miR-103 was co-transfected with circRNA_101505. The results showed that miR-103 mimic could significantly reversed circRNA_101505-mediated induction of apoptosis (Fig. 3d, e) and promotion of cisplatin toxicity in Hep3B-R and Huh7-R cells (Fig. 3f).

### MiR-103 confers cisplatin resistance in HCC cells by targeting NOR1

**Fig. 4:**
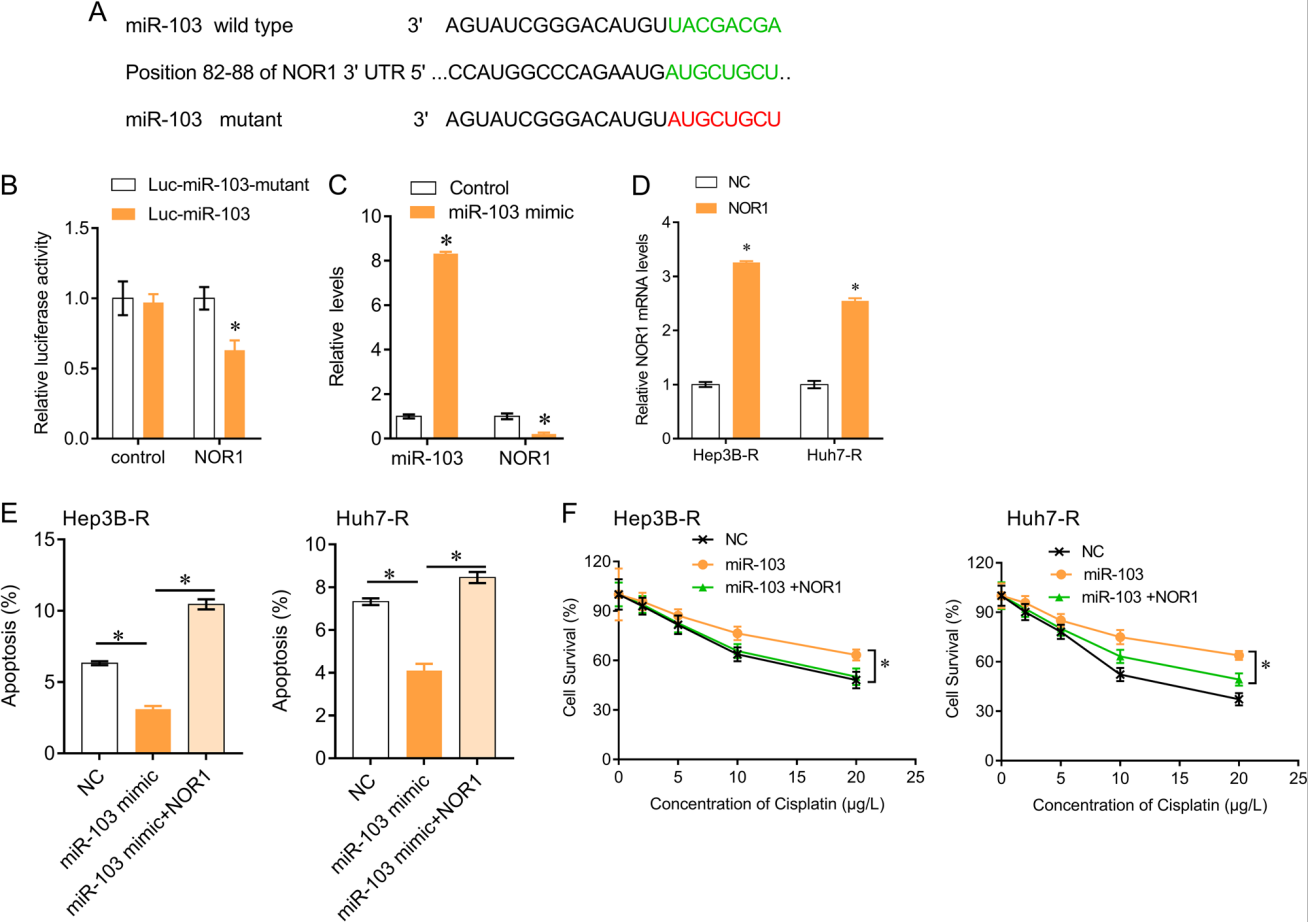
MiR-103 targets to NOR1 in HCC cells. **a** The predictive binding sites of miR-103 to 3′-UTR of NOR1 (green), the red color showed the mutant bases in binding site. **b** Hep3B-R cells were co-transfected Luc-miR-103 or Luc- miR-103 mutant with NOR1. Luciferase reporter gene assay was performed to measure luciferase activity. **c** QPCR for miR-103 and NOR1 in Hep3B-R cells after miR-103 mimics transfection. **d** QPCR for NOR1 in Hep3B-R and Huh7-R cells after NOR1 transfection. **e** NOR1 reversed miR-103-mediated reduction of apoptosis in Hep3B-R and Huh7-R cells. **f** NOR1 reversed miR-103-attenuated cisplatin toxicity in Hep3B-R and Huh7-R cells. *p < 0.05

**Fig. 5:**
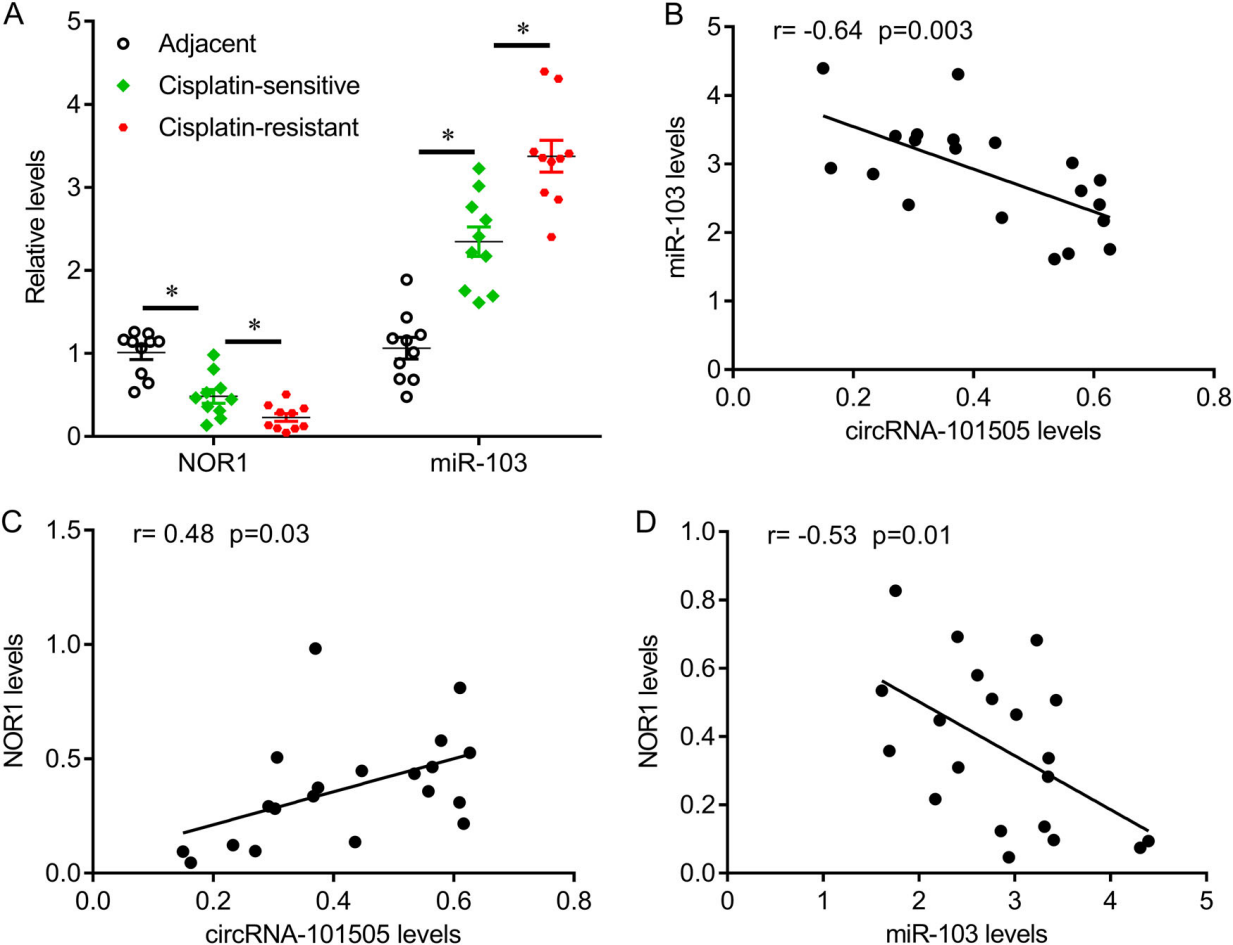
The relationship of circRNA_101505, miR-103 and NOR1 in patients with HCC. **a** Scatter plots illustrating qRT-PCR analysis of expression fold change for miR-103 and NOR1 in cisplatin-resistant and cisplatin-sensitive HCC tissues. **b** circRNA_101505 expression was negatively correlated with miR-103. **c** circRNA_101505 expression was positively correlated with NOR1. **d** miR-103 expression was negatively correlated with NOR1. *p < 0.05 vs control

We further investigated the role and downstream mechanism that miR-103 affects cisplatin resistance in HCC. NOR1 was a predictive target of miR-103 (Fig. 4a). MiR-103 wild-type and mutant sequences were constructed into luciferase reporter gene and co-transfected with NOR1, and luciferase assay confirmed that miR-103 targeted to NOR1 (Fig. 4a, b), and negatively regulated NOR1 expression (Fig. 4c). We expressed NOR1 in Hep3B-R and Huh7-R cells (Fig. 4d), and found that NOR1 could significantly reversed miR-103-mediated inhibition of apoptosis and suppression of cisplatin toxicity in Hep3B-R and Huh7-R cells (Fig. 4e, f). In addition, we also found that the levels of miR-103 were significantly increased and NOR1 was decreased in HCC tissues compared with adjacent tissues (Fig. 5a), and miR-103 levels were higher but NOR1 levels were lower in cisplatin-resistant HCC tissues than in the cisplatin-sensitive tissues (Fig. 5a). Pearson analysis showed that circRNA_101505 were negatively correlated with miR-103 (Fig. 5b) and positively correlated with NOR1 (Fig. 5c), and miR-103 was negatively correlated with NOR1 (Fig. 5d). These results demonstrate that circRNA_101505 sensitizes HCC cells to cisplatin through miR-103/NOR1 axis.

## Discussion

In this study, we found that circRNA_101505 downregulation is associated with a poor survival outcome and cisplatin resistance in HCC. Functional investigations revealed that circRNA_101505 overexpression inhibited HCC cell growth and sensitized HCC cells to cisplatin. Further experiments demonstrated that circRNA_101505 exhibited tumor suppressive effects by sponging miR-103, leading to NOR1 upregulation.

Cisplatin is a small-molecule platinum compound. Cisplatin-based chemotherapeutic regimens are one of the most widely used regimens for the treatment of various solid cancers, including liver cancer, cervical cancer and several others. The anticancer activity of cisplatin involves multiple mechanisms^10^. However, drug resistance of cisplatin limits its application and effectiveness. Cisplatin resistance may result from reduced drug accumulation inside cancer cells and drug inactivation by fast repairing of DNA lesions^11^. Several efforts have been made to minimize cisplatin resistance^12^. Recently, new evidence shows that circRNAs are associated with cisplatin resistance^13^. For example, upregulation of circPVT1 was observed in osteosarcoma tissues, serums and cisplatin-resistant cell lines, and was correlated with poor prognosis of patients with osteosarcoma. circPVT1 knockdown sensitizes osteosarcoma cells to cisplatin by decreasing ABCB1 expression^14^. The expression of circAKT3 was upregulated in cisplatin-resistant gastric cancer tissues and cells compared with cisplatin-sensitive samples, and was associated with aggressive characteristics and poor survival outcomes. CircAKT3 promotes DNA damage repair and inhibits apoptosis through PIK3R1 activation by sponging miR-198^15^. Chi et al. have reported that hsa_circ_0000285 expression is lower in patients with cisplatin-resistant bladder cancer, and acts as an independent prognostic factor for bladder cancer patient outcome^16^. In this study, we demonstrated that circRNA_101505 was downregulated in HCC tissues and associated with poor survival outcomes and cisplatin resistance in HCC. Gain-of-function experiments revealed that circRNA_101505 overexpression inhibited HCC cell proliferation, induced apoptosis and sensitized HCC cells to cisplatin in vitro and in vivo.

Further investigations showed that circRNA_101505 interacted with miR-103, and miR-103 mimics reversed circRNA_101505-mediated tumor suppressive effects. Noncoding RNAs, including miRNAs, lncRNAs and circRNAs, play an important role in the evolution and progression of drug resistance in cancers. The mechanisms are associated with drug transporter-related proteins, cell apoptosis-related proteins, DNA damage repair, and tumor microenvironment^17^. MiR-103 expression is upregulated in Adriamycin-resistant acute myeloid leukemia cells and confers K562 cells’ drug resistance via regulation of COP1 through PI3K/AKT signal pathway^18^. MiR-103 is controlled only by the MET oncogene and associated with gefitinib-induced apoptosis and epithelial-mesenchymal transition of non-small cell lung cancers in vitro and in vivo^19^. Furthermore, a recent study also demonstrates that circular RNA circARF3 acts as an endogenous miR-103 sponge to alleviate adipose inflammation by promoting mitophagy^20^.

In this study, we identified circRNA_101505 as a new interactive molecule of miR-103, also confirmed that NOR1 was a new downstream target of miR-103. NOR1 was cloned in our previous study and was involved in the development and progression of nasopharyngeal carcinoma^21,22^. Moreover, it inhibited proliferation and induced apoptosis via the MAPK activation in prostate cancer cells^23^. In hepatocellular carcinoma, we previously demonstrated that NOR1 increases the expression of growth factor receptor-bound protein 2 (Grb2) mRNA in the human hepatocellular carcinoma cells, and NOR1 enhanced monofunctional alkylating agent 5-(aziridin-1-yl)-2,4-dinitrobenzamide (CB1954)-induced cell killing in HCC cells, but this effect was reversed by stable transfection of Grb2 small hairpin RNA^24^. These results indicate that circRNA_101505 functions as a tumor suppressor by sponging miR-103, leading to NOR1 upregulation, and finally inhibits cell growth and sensitizes HCC cells to cisplatin.

In conclusion, our study revealed that circRNA_101505 is frequently inactivated in cisplatin-resistant HCC tissues and cell lines and associated with a poor survival outcome. CircRNA_101505 may sponge miR-103 and thereby promote NOR1 expression. Subsequently, this may suppress cell growth and sensitize HCC cells to cisplatin in vitro and in vivo. The significant inhibitory effects indicate circRNA_101505 as a potential therapeutic target for HCC treatment. Therefore, our findings provide significant evidence to further elucidate the therapeutic use of circRNA in HCC.

## Material and methods

### Human tissues collection

This project was approved by the Ethic Committee of The Third Xiangya Hospital of Central South University. Written informed consent was obtained from each patient. Hepatocellular carcinoma tissues (*N* = 120) and the adjacent tissues (*N* = 78) were collected from The Third Xiangya Hospital of Central South University between May 2008 and Nov 2018. Each sample was immediately flash-frozen in liquid nitrogen until RNA extraction.

### CircRNA microarray analysis

Total RNA was extracted from patients with cisplatin-resistant or cisplatin-sensitive HCC using the RNeasy Mini Kit (Qiagen, GmBH, Hilden, Germany) according to the manufacturer’s instructions. The adjacent tissues were used as control. Patients with cisplatin-resistant HCC were defined as those with persistent disease more than two months, and those with recurrent disease more than 2 months after completion of chemotherapy containing cisplatin. Patients with cisplatin-sensitive HCC were defined as those without local residual lesions or recurrence at 2 months after completion of chemotherapy containing cisplatin. Purified total RNA was quantified using the NanoDrop 2000 spectrophotometer. The total RNA was sent to Aksomics Co. Ltd. (Shanghai, China) to analyze circRNA expression profiles. Differentially expressed circRNAs were identified as fold change > 2 and adjusted p < 0.05.

### Cell culture

The cell lines used in the present study included Hep3B and Huh7 cells were cultured in Roswell Park Memorial Institute (RPMI) 1640 medium (Gibco) supplemented with 10% fetal bovine serum and maintained in a humidified atmosphere with 5% CO_2_ at 37 °C.

The cisplatin-resistant Hep3B (Hep3B-R) and Huh7 (Huh7-R) cells was established in our lab. The Hep3B or Huh7 cells were treated with the initial concentration of cisplatin (0.5 μg/L). The medium was changed once every 3 days. Each dose was maintained for 2 weeks, and then was doubled.

To test the cell survival and proliferation rates, Hep3B-R and Huh7-R cells were exposed to cisplatin at a series of concentrations (0, 2.5, 5, 10 and 20 μg/L) for 24 h or at 10 μg/L for 24 h.

### Cell transfection

circRNA_101505 overexpression plasmids (circRNA_101505-OE) and NOR1 expressed plasmids were constructed and purchased from (GenomediTech, Shanghai, China). miR-103 inhibitors and mimics were purchased from RiboBio (Guangzhou, China). Transfection was performed using Lipofectamine 3000 (LifeTechnologies, USA) according to the manufacturer’s instructions.

### Cell proliferation analysis

A BrdU Cell Proliferation Assay Kit (CST (Shanghai) Biological Reagents Company Limited, Shanghai, China) was used to measure the cell proliferation rate according to the manufacturer’s instructions. The cells were seeded in a 96-well plate and incubated overnight. The cells then were transfected circRNA_101505, miR-103 or NOR1, with co-treatment of cisplatin and cells were incubated for 24 h. Finally, 10 μM BrdU was added to the plate and cells were incubated for 4 h. The absorbance at 450 nm was read and recorded to calculate the relative proliferation rate. The experiment was done three times with triplicate samples.

### Cell apoptosis analysis

An Annexin V-FITC Apoptosis Detection Kit (CST (Shanghai) Biological Reagents Company Limited, Shanghai, China) was used for analysis of apoptosis according to the manufacturer’s instructions. Briefly, cells with indicated treatment were resuspended in 236 μl Annexin V-FITC binding buffer and then mixed well with 1 μl Annexin V-FITC and 13 μl Propidium Iodide for 30 min incubation. The cells were analyzed by using a BD FacScanto II flow cytometer (BD Biosciences, San Jose, CA). Unstained cells, and fluorescence minus one (FMO) controls were used for cytometry and gating set up.

### Quantitative PCR analysis

Total RNA was extracted with Trizol reagent. The expression of circRNA_101505 and NOR1 was measured by TaqMan Fast Advanced Master Mix (Thermo Scientific, Shanghai, China) according to manufacturers’ instructions. Expression of β-actin was used as an endogenous control. QPCR was performed at the condition: Hold 50 °C for 2 min, 95.0 °C for 20 s, and 40 circles of 95.0 °C for 1 s and 60 °C for 20 s in 7900HT Fast Real-Time PCR Instrument. The primers were used as following: circRNA_101505: forward, 5′-CCGAGTTCCTAAAAGCAGCC-3′; reverse, 5′-CCATCAGCAGTCCTAGGTCC-3′. β-actin: forward, 5′-CACACTGTGCCCATCTATGAGG-3′; reverse, 5′-TCGAAGTCTAGGGCGACATAGC-3′. miR-103 expression was measured by miRNA QPCR Master Mix (Agilent, Beijing, China) according to the manufacturer’s instructions.

### Luciferase reporter assay

The wild-type sequence and mutants in binding sites of circRNA_101505 was cloned downstream of FL reporter vector. Hep3B cells were seeded and cultured in 96-well plates for 24 h. The cells were co-transfected with FL reporter, Renilla luciferase reporter and miR-103 mimic for 48 h. Luciferase activity was measured using a Luciferase Reporter Assay Kit (BioVision Technologies, Inc, Exton, PA, USA). Relative luciferase activity was normalized to Renilla activity.

### RNA immunoprecipitation (RIP)

A Magna RIP™ RNA-Binding Protein Immunoprecipitation Kit (Merck Life Science (Shanghai) Co., Ltd. Shanghai, China) was used in this assay as previously describe (22234798). Briefly, Hep3B cells were fixed with formaldehyde, lysed with lysis buffer and sonicated. The supernatant was collected and incubated with a circRNA_101505-specific probes dynabeads (Invitrogen) mixture overnight at 30 °C. On the next day, the dynabeads was washed and incubated with 200 μl of lysis buffer. Finally, RNA was extracted from these complexes and was used for qPCR.

### Tumor xenograft in nude mice

Animal experiments were approved by the Ethical Committee for Animal Research of the Third Xiangya Hospital of Central South University. Ten nude mice (5 mice per group, male, 2 months old) were purchased from Animal Center of Central South University. The Hep3B-R cells transfected with circRNA_101505-OE was subcutaneously injected into mice. The tumor sizes were measured every week. The tumor volume was calculated using the formula: 0.5 × *L* × *W*^2^ where L and W are the long and short diameter of the tumor, respectively.

### Immunohistochemistry

The expression of Ki67 in tumor tissues from nude mice was analyzed by immunohistochemical analysis. Briefly, the tissues were fixed with 4% formaldehyde for 24 h, embedded and cut into 4-μm-thick section. The sections were treated with 10 mmol/l sodium citrate buffer and incubated with anti-Ki67 antibody (1: 200 dilution) overnight at 4 °C. The positive signaling was stained by using a Mouse and Rabbit Specific HRP/DAB (ABC) Detection IHC kit (Abcam Trading (Shanghai) Company Ltd., Shanghai, China), and counterstained with hematoxylin. The relative integral optical density (IOD) of positive signaling was obtained by ImageJ software.

### Statistical analysis

Data were analyzed in GraphPad Prism software (GraphPad Software Inc., La Jolla, CA). Overall survival analysis was performed by Kaplan–Meier curves and log-rank test for significance. Student’s t test with two tails was used to assess the statistical significance in two groups and one-way ANOVA with post hoc Bonferroni test were used in three or more groups. Correlations were analyzed by Pearson correlation test. *P* < 0.05 was considered statistically significant.
